# The *BAX* gene as a candidate for negative autophagy-related genes regulator on mRNA levels in colorectal cancer

**DOI:** 10.1007/s12032-016-0869-y

**Published:** 2016-12-29

**Authors:** Justyna Gil, David Ramsey, Elzbieta Szmida, Przemyslaw Leszczynski, Pawel Pawlowski, Marek Bebenek, Maria M. Sasiadek

**Affiliations:** 1 Department of Genetics, Wroclaw Medical University, 50-368 Wroclaw, Poland; 2 Department of Operations Research, Wroclaw University of Technology, 50-372 Wroclaw, Poland; 3 Department of Biology and Medical Parasitology, Wroclaw Medical University, 50-345 Wroclaw, Poland; 4 First Department of Surgical Oncology, Lower Silesian Oncology Center, 53-413 Wroclaw, Poland

**Keywords:** Autophagy, Apoptosis, Colorectal cancer, Relative expression

## Abstract

Autophagy is a catabolic process, which is involved in the maintenance of intracellular homeostasis by degrading redundant molecules and organelles. Autophagy begins with the formation of a double-membrane phagophore, followed by its enclosure, thus leading to the appearance of an autophagosome which fuses with lysosome. This process is highly conserved, precisely orchestrated and regulated by autophagy-related genes. Recently, autophagy has been widely studied in different types of cancers, including colorectal cancer. As it has been revealed, autophagy plays two opposite roles in tumorigenesis, as a tumor suppressor and a tumor enhancer/activator, and therefore is called a double-edge sword. Recently, interaction between autophagy and apoptosis has been found. Therefore, we aimed to study the mRNA levels of genes engaged in autophagy and apoptosis in colorectal cancer tissues. Colorectal cancer and adjacent healthy tissues were obtained from 73 patients diagnosed with primary colorectal cancer. Real-time PCR analysis employing Universal Probe Library was used to assess the expression of the seven following selected genes: *BECN1*, *UVRAG*, *ULK1*, *ATG13*, *Bif*-*1*, *BCL2* and *BAX*. For all but one of the tested genes, a decrease in expression was observed. An increase in expression was observed for *BAX. BAX* expression decreases consistently from early to more advanced stages. High expression of *BAX* was strongly associated with negative *UVRAG* expression. The high expression of the *BAX* gene seems to be a negative regulator of autophagy in colorectal cancer cells. The relative downregulation of autophagy-related genes was observed in colorectal cancer samples.

## Introduction

Carcinogenesis is a complex, multistep process during which acquired genomic alterations may lead to chromosomal, microsatellite and epigenetic instability and thus result in cancer progression [1]. Cancers are the second leading cause of morbidity and mortality worldwide [2]. Colorectal cancer (CRC) is one of the most common cancers in developed regions, such as Australia, Europe and North America, and the leading cause of cancer-related deaths [2]. Its incidence in these regions is high (about 55% of all cancer cases) and ranges from the second to third (depending on population ethnicity) most common type of cancer among both sexes [2]. Most CRCs are sporadic, and individual susceptibility to disease is determined by: (1) environmental factors, such as occupational exposure, dietary habits and lack of physical activity, as well as (2) genetic makeup, including polymorphic variants in genes responsible for cellular metabolism and DNA repair (low-risk variants) [3, 4]. Despite immense progress in knowledge of genetic and environmental factors in CRC etiology, along with new treatment approaches which have been recently introduced into clinical practice, this cancer is usually diagnosed at a late stage of disease and thus 5-year overall survival is not frequent [5]. Recently, macroautophagy (hereafter autophagy) has emerged as a promising independent prognostic molecular biomarker and a potential target in cancer therapy [6]. Autophagy is a catabolic process enabling the maintenance of normal cell homeostasis by degrading redundant molecules and organelles (“self-eating”), but is also responsible for intracellular recycling, e.g., reuse of amino acids from degraded proteins [7]. Briefly, a cargo designed for degradation is engulfed by a double-membraned vesicle, called an autophagosome, which fuses with lysosome and thus its content is decomposed by acidic enzymes [8]. Autophagy is a fundamental cellular process which is highly conserved from yeasts to humans, and many yeast genes involved in autophagy have human orthologs (AuTophaGy related; *ATG*). Autophagy, as a defense process, is usually upregulated in cells under conditions of stress, e.g., starvation [8]. Thus, the energy essential for maintaining basic cellular functions may be acquired by the process of degrading proteins or organelles which are less necessary for cell survival (pivotal structures remain intact) in the process of autophagy [8]. The induction of autophagy is regulated by a variety of genes, including *ULK1*, *ATG13*, *UVRAG*, *Bif*-*1* and *BECN1*. The following steps of autophagy result in the elongation and maturation of autophagosomes [8]. Eventually, autophagosomes fuse with lysosomes, thus forming autophagolysosomes and their content may be degraded by hydrolases [8].

Recently, autophagy has been extensively studied in different types of tumors, e.g., breast, pulmonary, prostate, brain and colorectal [7]. Up to now, autophagy in carcinogenesis has been described as a double-edged sword because of its dual function. On the one hand, autophagy protects normal cells against neoplastic transformation by maintaining intracellular homeostasis, but, on the other hand, may result in cancer cells being more likely to survive than normal cells under adverse circumstances, such as hypoxia and starvation, as well as during anticancer therapy [7, 9]. To date, the results of many studies on autophagy in CRC are conflicting and inconclusive; thus, its function in CRC development and progression remains unclear. Recently, a complex interaction between autophagy and apoptosis was reported. However, studies have shown conflicting results [10].

Because of inconclusive research data, we have focused on the mRNA expression levels of five genes involved in the induction of autophagy: *BECN1*, *UVRAG*, *ULK1*, *ATG13* and *Bif*-*1* and two genes involved in apoptosis: the antiapoptotic *BCL2* and the proapoptotic *BAX*. These expression levels were observed in both colorectal cancer cells and paired relatively normal, adjacent tissue.

## Materials and methods

### Patients

Surgical samples of tissue were obtained from 73 patients with primary colorectal cancer admitted to the First Department of Surgical Oncology, Lower Silesian Oncology Center, Wroclaw, Poland, between 2010 and 2013. The mean age of the patients was 64.274 with a standard deviation of 11.066 (ranging from 35 to 88 years). The study group was evenly split with respect to sex: 49.32% female (36 of 73) and 50.68% male (37 of 73). All the tumors were classified as adenocarcinomas and were examined by two independent pathologists and classified according to the TNM classification stage criteria. Forty-six of the tumors (63%) were located on the left (descending colon, sigmoid colon and rectum) and 27 (37%) on the right (cecum and ascending colon). Patients included in the studies had no family history in regard to hereditary cancer syndromes. None of the patients received radiation or chemotherapy preoperatively. Detailed characteristics of the patients are shown in Table 1. Written informed consent was obtained from all patients before enrollment. The study design was accepted by the Wroclaw Medical University Ethical Committee (approval number KB-822/2012).

**Table 1:** Clinical and pathological characteristics of CRC patients

Variable	Total (%)
Gender	
Female	36 (49.3)
Male	37 (50.7)
Age	
<50	7 (9.6)
>50	66 (90.4)
Primary tumor (T)	
T1	0
T2	9 (12.3)
T3	34 (46.6)
T4	30 (41.1)
Regional lymph nodes (N)	
Nx	2 (2.7)
N0	8 (11)
N1	40 (54.8)
N2	20 (27.4)
N3	3 (4.1)
Distant metastasis (M)	
Mx	1 (1.4)
M0	65 (89)
M1	7 (9.6)
TNM classification	
I	3 (4.1)
II	6 (8.2)
III	57 (78.1)
IV	7 (9.6)
Tumor location	
Right colon	27 (37)
Left colon	46 (63)

### Methods

Fresh tumor specimens and adjacent noncancerous tissue were collected in 5 ml of RNA later (Qiagen) and stored at −20 °C. Isolation of RNA was performed with the TriPure Isolation Reagent (Roche Diagnostics) following the standard protocol. The concentration, quality, purity and integrity of RNA were determined using Experion RNA StdSens Chips (Bio-Rad) for the Experion Automated Electrophoresis System (Bio-Rad). RNA samples with concentration over 100 ng/μl and RNA quality indicator (RQI) over 5 were qualified for further analysis. One microgram of total RNA from each sample was used for cDNA synthesis by reverse transcription using the Transcriptor First Strand cDNA Synthesis Kit (Roche Diagnostics) with standard random hexamer priming according to the manufacturer’s instructions. cDNA was either immediately used for PCR setup or stored at −20 °C. The expression of target genes was normalized relative to three chosen reference genes *GAPDH* (GeneID: 2597), *PPIA* (GeneID: 5478) and *RPLP0* (GeneID: 6175). A RealTime Ready Custom Panel 96-32+ (Roche Diagnostics) layout for 96 reactions in a dried-down format in 96-well plates was applied to carry out a real-time PCR assay. The custom panel assays contained target-specific primers and a matching probe from the Universal Probe Library (UPL). The RealTime Ready assays comply with the Minimum Information for Publication of Quantitative Real-time PCR Experiments (MIQE) guidelines [11]. The real-time PCR mix was prepared from cDNA preparations according to the standard procedures as given by the manufacturer using the LightCycler 480 Probes Master (Roche Diagnostics) by the LightCycler 480 machine. The LightCycler 480 software, version 1.5.1, and the sample editor content *.txt file (Roche Diagnostics) were used for sample setup, real-time PCR analysis, as well as calculation of the relative *C*_t_ values.

### Statistical analysis

The $2^{{ - {\Delta \Delta }C_{\text{t}} }}$ method, as described by Livak and Schmittgen [12], was applied to assess the relative difference in expression between healthy and cancer cells. Student’s *t* test was used to compare means for two groups, since the group size is sufficiently large. The significance of associations was determined using Spearman’s correlation coefficient, which is more robust to deviations from linear relationships and can be applied in conjunction with ordinal variables (e.g., grades). In addition to the results from these classical tests, the Benjamini–Hochberg procedure for multiple testing was applied.

## Results

### Associations with differences in expression levels between tumor cells and healthy cells

The ranking of gene expression levels from the highest to the lowest values based on the delta *C*_t_ method is as follows: in relatively healthy, adjacent normal mucosa *Bif*-*1*, *BECN1*, *ATG13*, *BAX*, *BCL2*, *ULK1*, *UVRAG*, in cancer tissue *Bif*-*1*, *BECN1*, *BAX*, *ATG13*, *BCL2*, *ULK1*, *UVRAG*, see Table 2.

**Table 2:** Ranking of genes according to expression

Gene	*R*_N_	*Δ*_N_	95% CI	*R*_T_	*Δ*_T_	95% CI
Bif-1 (SH3GLB1)	1	4.2075	4.0660, 4.3489	1	4.5973	4.4431, 4.7515
BECN1	2	4.6504	4.4952, 4.8056	2	5.1683	5.0235, 5.3131
ATG13	3	6.5206	6.3121, 6.7290	4	6.5064	6.3309, 6.6819
BAX	4	6.6249	6.3831, 6.8667	3	6.1592	5.9480, 6.3705
BCL2	5	8.3581	8.0622, 8.6541	5	9.5103	9.1518, 9.8687
ULK1	6	9.0432	8.6375, 9.4490	6	9.5489	9.2179, 9.8799
UVRAG	7	9.2674	8.9203, 9.6145	7	10.1138	9.8233, 10.4044

*Δ*_N_ and *Δ*_T_ denote the delta scores for normal and tumor cells, respectively. *R*_N_ and *R*_T_ denote the rankings according to these scores for normal and tumor cells, respectively

The mRNA relative expression levels of *BCL2*, *BECN1*, *UVRAG* and *Bif*-*1* cancer cells were lower than those in adjacent colon tissues, ranked according to the significance of the relative change in expression (*p* < 0.05), see also Table 3. The changes in the mRNA relative expression levels of *ULK1* were not significant, see Table 3. The mRNA relative expression level of *BAX* was higher in cancer cells than in adjacent colon tissues (*p* < 0.05), see Table 3.

**Table 3:** Ranking of genes according to mean relative fall in expression

Pos.	Gene	ΔΔ	95% CI	2^−ΔΔ^	95% CI
1	BCL2	−1.1521	−1.6196, −0.6847	2.2224	1.6073, 3.0729
2	UVRAG	−0.8464	−1.2991, −0.3938	1.7980	1.3138, 2.4607
3	BECN1	−0.5179	−0.7302, −0.3057	1.4319	1.2360, 1.6589
4	ULK1	−0.5057	−1.0271, 0.0158	1.4198	0.9891, 2.0379
5	SH3GLB1	−0.3899	−0.5991, −0.1806	1.3103	1.1334, 1.5148
6	ATG13	0.0142	−0.2594, 0.2878	0.9902	0.8191, 1.1970
7	BAX	0.4657	0.1446, 0.7868	0.7241	0.5796, 0.9047

### Location, T, N, M, advancement

“T” was negatively correlated with expression levels at *BAX*: (higher T correlated with higher scores, i.e., lower expression) Spearman’s correlation coefficient *R* = 0.247 (*p* = 0.035). Moreover, the expression of *BAX* was lower (in comparison with adjacent, relatively normal tissue) among those patients with distant metastasis *M* = 1 (*p* = 0.047). However, these differences are not significant when the Benjamini–Hochberg procedure is applied. The expression of *BAX* was higher among tumors located on the left (*p* = 0.014), but this was not significant when the Benjamini–Hochberg procedure was taken into account.

The fall in expression levels of various genes was generally positively correlated with each other. The one exception was *BAX*. An increase in the expression level of this gene was associated with a fall in the expression level of *UVRAG*. The following correlation was significant: Spearman’s correlation coefficient *R* = −0.299 (*p* = 0.010).

There were no other significant associations between the location, T, N, M staging nor degree of advancement of the tumor and the difference between the expression levels in tumor and healthy cells of any gene.

### Age

Age was positively correlated with the fall in expression levels between healthy and tumor cells of the four following genes: *BECN1* Spearman’s correlation coefficient *R* = 0.343 (*p* = 0.003), *UVRAG* Spearman’s correlation coefficient *R* = 0.274 (*p* = 0.019), *ATG13* Spearman’s correlation coefficient *R* = 0.271 (*p* = 0.021) and *ULK1* Spearman’s correlation coefficient *R* = 0.274 (*p* = 0.024). The association between age and the fall in expression of *BECN1* remains significant when the Benjamini–Hochberg procedure for multiple testing is applied.

### Sex

Sex is not significantly associated with the difference in expression levels between healthy and tumor cells of any gene.

## Discussion

Genetic mutations leading to the activation of protooncogenes and/or loss of functioning of tumor suppressor genes may lead to the deregulation of various cellular pathways, including autophagy, and thus to cancer formation [13]. Autophagy is an intracellular mechanism responsible for defense against cellular stress [14]. However, its role in cancer initiation, tumor growth, anticancer therapy and treatment still remains an unanswered question [14].

In our study, we have shown relative downregulation of all but one of the examined autophagy-related genes, along with antiapoptotic *BCL2*, whereas proapoptotic *BAX* was relatively upregulated. We have observed its higher expression in the early stages of CRC in comparison with normal tissue. However, *BAX* expression successively decreases as a cancer progresses and is the lowest in patients with distant metastasis. Our results are in agreement with the observations published by Jansson and Sun [15]. They examined the protein expression level of BAX in normal colorectal mucosa, as well as in primary colorectal adenocarcinomas from early to advanced stages, including cases with metastases to regional lymph nodes. They reported more intense expression in primary tumors in comparison with normal tissue, but in metastatic CRC samples, lower expression levels have been observed [15]. Similar results have been obtained by Cobanoglu et al., who examined expression levels of BAX and AIF (apoptosis-inducing factor). BAX staining levels were markedly higher in adenomas and carcinomas than in normal mucosa. Moreover, the BAX level was higher in carcinomas than in adenomas [16].

Therefore, we may conclude that during the early stages of CRC carcinogenesis apoptosis is more prone to occur than autophagy, while during tumor progression an accumulation of genetic alterations may disturb the process of apoptosis and thus contribute to tumor progression and promotion.

We have observed a statistically significant correlation between a high expression of *BAX* and a decrease in expression of *UVRAG.* UVRAG is a well-known protein involved in autophagy initiation, through interaction with BECN1, as well as in the maturation of autophagosomes [8]. Recently, UVRAG has been reported as a crucial factor in apoptosis. Yin et al. [17] found that UVRAG possesses both autophagic and antiapoptotic properties mediated by its direct interaction with BAX in the cytosol, as confirmed by coimmunoprecipitation studies. These researchers have formulated the hypothesis that UVRAG exerts its cytoprotective function by controlling the localization of the BAX protein through interaction with this protein and inhibits translocation of BAX to the mitochondria and therefore prevents apoptosis [17]. Increased expression of UVRAG has been observed in cells exposed to stress, such as chemotherapy and/or UV radiation. The influence of the underexpression of UVRAG on anticancer therapy has been studied in experiments in which UVRAG expression has been inhibited by specific short hairpin RNAs (sshRNAs) transfection [17]. A decreased index of autophagy and increased level of apoptosis were detected [17]. Therefore, the authors suggested that decreased UVRAG activity directly influences BAX-induced apoptosis in cancer cells. The authors also showed that the antiapoptotic activity of UVRAG does not affect BAX expression [17]. However, UVRAG does not influence apoptosis induced by other proapoptotic proteins, such as Bad or Bid. Moreover, its direct role in the regulation of apoptosis seems to be an independent event, besides its proautophagic function [17]. Thus, it was assumed that in tumor cells UVRAG plays a central role in the modulation of apoptosis in response to stressful conditions (UVRAG-BAX complex) as a negative regulator and autophagy (UVRAG-BECN1 complex) as a positive regulator [17]. In our study, an elevated level of mRNA in *BAX* was shown to be associated with downregulation of the mRNA levels of *UVRAG.* Hence, we hypothesized that the promotion of apoptosis may influence the expression of *UVRAG* and therefore counteracts the induction of autophagy in CRC cells. Consequently, we conclude that high *BAX* expression may be a negative regulator of *UVRAG* gene expression.

Among the analyzed genes, we found that *Bif*-*1* (BAX-interacting factor 1) expression was the highest, both in normal and in cancer tissues. Bif-1 is also known as SH3GLB1 (SH3 domain GBR2-like endophilin B1) and belongs to the endophilin protein family [18]. Bif-1 was identified as a BAX-binding protein and a necessary factor in the promotion of apoptosis [19]. It has been proven that the loss of Bif-1 inhibits the following: (1) BAX/Bak conformational activation, (2) release of cytochrome c and (3) caspase activation in response to intrinsic signals of death [19]. Overexpression of Bif-1 stimulates BAX and thus stimulates apoptosis. It has been hypothesized that Bif-1 may be a new type of BAX activator controlling apoptosis in the mitochondrial pathway [20]. Moreover, Bif-1 is also involved in autophagy and its complex with BECN1 in conjunction with UVRAG is required for the induction of autophagosome formation [19]. Coppola et al. [20] found decreased levels of both Bif-1 mRNA and protein in CRC tissues. These results are in agreement with our results. The *Bif*-*1* gene is located on the short arm of chromosome 1 (locus: 1p22). This region is frequently deleted in many human cancers, including CRC [21–24]. Therefore, it has been proposed that *Bif*-*1* is a tumor suppressor gene. Loss of Bif-1 functioning may suppress apoptosis, as well as autophagy [20].

The fact that the *Bif*-*1* gene had the highest level of expression among all the genes tested in our study may be explained by the fact that this protein is involved in two independent intracellular pathways connected with cell death, namely apoptosis and autophagy. We found decreased *Bif*-*1* mRNA levels in CRC samples, and therefore, we hypothesize that this decrease may result in the suppression of autophagy. However, as we also observed increased *BAX* gene expression in CRC samples, it can be argued that upregulation of apoptosis in CRC cells may be a driving force which downregulates autophagy and *Bif*-*1* downregulation leads to its inhibition.

One of the most important proteins engaged in the initiation of autophagy is BECN1 (beclin 1) encoded by the *BECN1* gene located on the long arm of chromosome 17 (locus 17q21.31). It has been hypothesized that *BECN1* acts as tumor suppressor gene, because of its frequent deletion in a variety of tumors such as breast, ovarian and prostate [25–27]. We found its expression level to be average in both tumor and normal tissue, with the level of expression being lower in tumor samples than in healthy tissue. Interestingly, the results of other authors are conflicting, as some studies found an increased BECN1 protein level in CRC samples [28–30], while some found a decreased level [31, 32]. Hence, its role in CRC pathogenesis remains unclear and needs to be elucidated by further analysis. We would like to emphasize that the most common methods used for the evaluation of BECN1 expression are immunohistochemistry (IHC) and Western blot. Both methods are semiquantitative, and it is difficult to found direct relationships between protein and mRNA levels, because of complex post-transcriptional and post-translational modifications [33].

Intriguingly, in our research we found that mRNA levels of *BECN1* and *UVRAG* genes are positively correlated with age, as older people exhibited higher expressions of both of them in normal tissue. Higher levels of the expression of autophagy regulators responsible for the induction of autophagy in the normal tissue of older people may be explained by the age-related failure of lysosomal hydrolases and the ineffectiveness of autolysosomes (accumulation of autophagic vacuoles), which cause autophagic activity to decline [34]. Therefore, in older people’s cells the accumulation of redundant molecules may stimulate higher levels of expression of genes responsible for the induction of autophagy (positive feedback).

We also observed medium expression levels of the *BCL2* gene and lower expression levels in CRC samples. The BCL2 gene negatively regulates two cell death pathways: apoptosis and autophagy [35]. As we found an elevated level of *BAX* and decreased level of *BCL2*, we suggest that in the early stages of CRC tumorigenesis, apoptosis is more prone to occur than autophagy. However, in metastatic samples a decrease in the expression level of *BAX* is surprisingly not accompanied by an increase in the expression of autophagy-related genes. This phenomenon should be studied more thoroughly.

We have found a complex correlation between two pathways connected with cell death. Autophagy, along with apoptosis, is responsible for normal cell development during morphogenesis and for maintaining intracellular homeostasis, as well as cell death in mature organisms [36]. The interaction between both pathways is critical for the cell life cycle. However, to date the studies published on this interaction have shown conflicting results. Some proteins, such as BECN1, UVRAG, ULK1, BCL2 and BAX, have revealed a dual role and may regulate both autophagy and apoptosis [37, 38]. In this research, we have found that the genes engaged in the induction of autophagy *ULK1* and *UVRAG* have the lowest expression levels in both cancer and normal tissue. Medium to high expression of mRNA was found in *BCL2*, *BECN1*, *ATG13* and *BAX*. The highest expression was found in *Bif*-*1*. Further functional analysis is needed to elucidate how these two pathways (autophagy and apoptosis) are interdependent.

Summarizing, our studies enable us to formulate the hypothesis that high mRNA expression of the proapoptotic *BAX* gene may play the role of a negative regulator of autophagy in CRC development.

## References

[1] Torre LA, Bray F, Siegel RL, Ferlay J, Lortet-Tieulent J, Jemal A (2015). Global cancer statistics, 2012. CA Cancer J Clin.

[2] Diaz-Cano SJ (2012). Tumor heterogeneity: mechanisms and bases for a reliable application of molecular marker design. Int J Mol Sci.

[3] Tomlinson IP, Dunlop M, Campbell H, Zanke B, Gallinger S (2010). COGENT (COlorectal cancer GENeTics): an international consortium to study the role of polymorphic variation on the risk of colorectal cancer. Br J Cancer.

[4] Haggar FA, Boushey RP (2009). Colorectal cancer epidemiology: incidence, mortality, survival, and risk factors. Clin Colon Rectal Surg.

[5] Brenner H, Kloor M, Pox CP (2014). Colorectal cancer. Lancet.

[6] Yang ZJ, Chee CE, Huang S, Sinicrope FA (2011). The role of autophagy in cancer: therapeutic implications. Mol Cancer Ther.

[7] Choi AM, Ryter SW, Levine B (2013). Autophagy in human health and disease. N Engl J Med.

[8] Yang Z, Klionsky DJ (2010). Mammalian autophagy: core molecular machinery and signaling regulation. Curr Opin Cell Biol.

[9] White EJ, Martin V, Liu JL, Klein SR, Piya S, Gomez-Manzano C, Fueyo J, Jiang H (2011). Autophagy regulation in cancer development and therapy. Am J Cancer Res.

[10] Bincoletto C, Bechara A, Pereira GJ (2013). Interplay between apoptosis and autophagy, a challenging puzzle: new perspectives on antitumor chemotherapies. Chem Biol Interact.

[11] Bustin SA, Benes V, Garson JA (2009). The MIQE guidelines: minimum information for publication of quantitative real-time PCR experiments. Clin Chem.

[12] Livak KJ, Schmittgen TD (2001). Analysis of relative gene expression data using real-time quantitative PCR and the 2^−ΔΔCt^ method. Methods.

[13] Su Z, Yang Z, Xu Y, Chen Y, Yu Q (2015). Apoptosis, autophagy, necroptosis, and cancer metastasis. Mol Cancer.

[14] Macintosh RL, Ryan KM (2013). Autophagy in tumour cell death. Semin Cancer Biol.

[15] Jansson A, Sun XF (2002). Bax expression decreases significantly from primary tumor to metastasis in colorectal cancer. J Clin Oncol.

[16] Cobanoglu B, Ceyran AB, Simsek M, Şenol S (2015). Immunohistochemical analysis of Bax and AIF in colorectal tumors. Int J Clin Exp Med.

[17] Yin X, Cao L, Kang R (2011). UV irradiation resistance-associated gene suppresses apoptosis by interfering with BAX activation. EMBO Rep.

[18] Takahashi Y, Meyerkord CL, Wang HG (2009). Bif-1/endophilin B1: a candidate for crescent driving force in autophagy. Cell Death Differ.

[19] Takahashi Y, Coppola D, Matsushita N (2007). Bif-1 interacts with Beclin 1 through UVRAG and regulates autophagy and tumorigenesis. Nat Cell Biol.

[20] Coppola D, Khalil F, Eschrich SA, Boulware D, Yeatman T, Wang HG (2008). Down-regulation of Bax-interacting factor-1 in colorectal adenocarcinoma. Cancer.

[21] Balakrishnan A, von Neuhoff N, Rudolph C (2006). Quantitative microsatellite analysis to delineate the commonly deleted region 1p22.3 in mantle cell lymphomas. Genes Chromosomes Cancer.

[22] Walker GJ, Indsto JO, Sood R (2004). Deletion mapping suggests that the 1p22 melanoma susceptibility gene is a tumor suppressor localized to a 9-Mb interval. Genes Chromosomes Cancer.

[23] Liang JW, Shi ZZ, Shen TY (2014). Identification of genomic alterations in pancreatic cancer using array-based comparative genomic hybridization. PLoS ONE.

[24] Knösel T, Petersen S, Schwabe H, Schlüns K, Stein U, Schlag PM, Dietel M, Petersen I (2002). Incidence of chromosomal imbalances in advanced colorectal carcinomas and their metastases. Virchows Arch.

[25] Qu X, Yu J, Bhagat G (2003). Promotion of tumorigenesis by heterozygous disruption of the beclin 1 autophagy gene. J Clin Invest.

[26] Liang C, Feng P, Ku B (2006). Autophagic and tumour suppressor activity of a novel Beclin1-binding protein UVRAG. Nat Cell Biol.

[27] Miracco C, Cosci E, Oliveri G (2007). Protein and mRNA expression of autophagy gene Beclin 1 in human brain tumours. Int J Oncol.

[28] Ahn CH, Jeong EG, Lee JW, Kim MS, Kim SH, Kim SS, Yoo NJ, Lee SH (2007). Expression of beclin-1, an autophagy-related protein, in gastric and colorectal cancers. APMIS.

[29] Chen Z, Li Y, Zhang C, Yi H, Wu C, Wang J, Liu Y, Tan J, Wen J (2013). Downregulation of Beclin 1 and impairment of autophagy in a small population of colorectal cancer. Dig Dis Sci.

[30] Zhang MY, Gou WF, Zhao S, Mao XY, Zheng ZH, Takano Y, Zheng HC (2014). Beclin 1 expression is closely linked to colorectal carcinogenesis and distant metastasis of colorectal carcinoma. Int J Mol Sci.

[31] Li ZD, Chen B, Wu YQ (2008). The expression of human tumor suppressor gene beclin 1 is down-regulated in gastric and colorectal cancer. Prog Biochem Biophys.

[32] Chang YT, Tseng HC, Huang CC, Chen YP, Chiang HC, Chou FP (2011). Relative down-regulation of apoptosis and autophagy genes in colorectal cancer. Eur J Clin Invest.

[33] Greenbaum D, Colangelo C, Williams K, Gerstein M (2003). Comparing protein abundance and mRNA expression levels on a genomic scale. Genome Biol.

[34] Cuervo AM (2008). Autophagy and aging: keeping that old broom working. Trends Genet.

[35] Rikiishi H (2012). Novel Insights into the Interplay between Apoptosis and Autophagy. Int J Cell Biol.

[36] Levine B, Klionsky DJ (2004). Development by self-digestion: molecular mechanisms and biological functions of autophagy. Dev Cell.

[37] Mukhopadhyay S, Das DN, Panda PK, Sinha N, Naik PP, Bissoyi A, Pramanik K, Bhutia SK (2015). Autophagy protein Ulk1 promotes mitochondrial apoptosis through reactive oxygen species. Free Radic Biol Med.

[38] Mukhopadhyay S, Panda PK, Sinha N, Das DN, Bhutia SK (2014). Autophagy and apoptosis: where do they meet?. Apoptosis.

